# 20 Years of European Society for Traumatic Stress

**DOI:** 10.3402/ejpt.v4i0.21368

**Published:** 2013-06-06

**Authors:** Brigitte Lueger-Schuster

In 1993, the European Society for Traumatic Stress (ESTSS) was founded by a group of men from all over Europe. At the time, 20 years ago, Europe was shaken by a war in southeast Europe. The Berlin Wall had fallen, the eastern countries of the continent were recovering from the consequences of problematic regimes, and the European Union was smaller than it is today. The continent of Europe was in some areas very rich and well developed; in other regions, it was poor and suffering from ongoing conflicts. This thematic cluster of papers has contributions from almost all past presidents of ESTSS, since the start of ESTSS in 1993 up to its 20th anniversary in 2013. Erica van der Schrieck-de Loos has written a memorial contribution given the sad loss of her father Walter de Loos. Bas Schreuder was unable to contribute but sent his congratulations and has explained his connectedness with ESTSS.

Erica van der Schrieck-de Loos cites an opening speech of her father, Wolter de Loos (the first president of ESTSS), in which he reflected about the victims, the victor, and the perpetrator (van der Schrieck-de Loos, 2013) related with the European history. The two World Wars were initiated by European states and the main battlefields were within the continent. European people have encountered situations full of traumatic features; generations have coped in their own specific ways, often by denying psychological consequences. Roderick Ørner (2013) describes the roots of ESTSS in his contribution, also stating that Europe was well trained in coping with wars, gross violations of human rights, and disaster. The emotional scars were mostly kept secret; only a few mental health professionals considered traumatic reactions when they treated individuals. But, some people had taken care of traumatized people, namely holocaust survivors, veterans, civilian victims of war, refugees, victims of sexual abuse and violence, children suffering from neglect, and survivors of disaster. These people met each other at conferences, read papers from colleagues in the United States (the DSM had already classified PTSD as a diagnosis), and lobbied for their patients. There had been a long tradition of interest in traumatic stress, known as traumatic neuroses, but these interests were cultivated in islands within the fields of psychiatry, psychotherapy, and clinical psychology. The topic was not yet broadly acknowledged within the field of mental health care. However, this was a discovery period as Stuart Turner writes in his contribution (Turner, 2013).

ESTSS started with inspiring conferences, the first one took place in Lincoln (Ørner, 2013). Integrative personalities like Wolter de Loos, Atle Dyregrov, and Lars Weisaeth were sitting in the background and mentoring the group that formed ESTSS. In the beginning, there had been contact with the Society of Traumatic Stress Studies (STSS), now the International Society of Traumatic Stress Studies (ISTSS), and the founding of ESTSS was not free from conflict with STSS. Berthold Gersons addresses these issues and the current cooperation of ESTSS and ISTSS (Gersons, 2013).

Most of the past presidents met in Bergen in 1993, the founding place of ESTSS. This was the case for Dean Ajduković and Ulrich Schnyder. Dean Ajduković, from the University of Zagreb, came from a country involved in a horrible civil war. In his contribution, he reflects on the introduction of the notion of social context of collective trauma, an immediate influence of the war situation in former Yugoslavia (Ajduković, 2013). Ulrich Schnyder, from Switzerland, found his way into psychotraumatology without this background of war; he found his professional home by looking out for a place to share interests with colleagues and identified ESTSS. Ulrich's contribution is about the development of the trauma field; he stresses organizational issues and “mutual learning globally” (Schnyder, 2013). Jonathan Bisson was also in Bergen in 1993 and was inspired by this conference. He has continued to attend ESTSS conferences since then, regularly presenting papers on evidence-based practice, the topic of his contribution to the thematic cluster. Further, Jonathan made the first practical steps to change ESTSS into an umbrella organization. Miranda Olff, our first female president, followed in his footsteps. She developed ESTSS into a “big player” by raising the number of members to almost 2,500. Miranda's contribution describes the process of restructuring and how ESTSS has grown. Together with Jonathan, she led the two TENTS-projects, which she also describes in her contribution. Miranda also established the *European Journal for Psychotraumatology* (EJPT), our journal.

Finally, I describe some professional steps in my own career, including a variety of ones related to traumatic stress. I use my own experience to reflect on the further development of ESTSS as a multilingual and culturally sensitive society for all matters concerning psychotraumatology. I consider that the integrative capacity of ESTSS, with the diverse European background in history, culture, scientific approaches, and languages will enrich our future activities and bring its services closer to individuals in need. This richness will be the contribution of ESTSS to the Global project that ISTSS has launched.

ESTSS has developed into a large society for professionals. Trauma, PTSD, and psychotraumatology have moved closer to the center of mental health. Leaders of the global field have a European background. Psychotraumatology has become a field of interest that attracts many people. ESTSS and its past presidents have worked hard to achieve the current status. I am deeply grateful for their pioneering work.

As the current president of ESTSS, it was my wish to celebrate the 20th birthday of ESTSS. Twenty years is an age when the first biographical notes can be put down. I, therefore, invited our past presidents to contribute to an ESTSS birthday edition of EJPT. Their answers came immediately, positive and enthusiastic.

The papers of all past presidents stress one recurrent theme: ESTSS is a professional home, built on knowledge, the sharing of experiences, a very active engagement in the field of psychotraumatology and friendship. Dealing with trauma is sometimes hard, frightening, and stressful. Having a professional home that includes high quality friendship is fantastic, especially when one is overwhelmed or in need of advice. I hope that ESTSS will retain its special qualities, for at least the next 20 years.

*Brigitte Lueger-Schuster* ESTSS president, 2011–2013 Guest Editor
